# Adverse Childhood Events, Resilience, and Psychopathology

**DOI:** 10.3390/medicina61122138

**Published:** 2025-11-30

**Authors:** Andreea-Mihaela Militaru, Simona Trifu, Amelia Damiana Trifu, Dana Galieta Minca

**Affiliations:** 1 Department of Clinical Psychology, Faculty of Psychology and Educational Sciences, University of Bucharest, 050663 Bucharest, Romania; 10andreea10.am@gmail.com; 2 Department of Clinical Neurosciences, “Carol Davila” University of Medicine and Pharmacy, 020021 Bucharest, Romania; 3 Department of General Medicine, “Carol Davila” University of Medicine and Pharmacy, 020021 Bucharest, Romania; ameliatrifu2002@gmail.com; 4 Department of Complementary Sciences, “Carol Davila” University of Medicine and Pharmacy, 020021 Bucharest, Romania; dana.minca@umfcd.ro

**Keywords:** adverse childhood experiences, psychiatric disorders, psychopathology, resilience, early maladaptive schemas, life history-based strategies

## Abstract

*Background and objectives*: Adverse childhood experiences increase the predisposition to developing psychiatric disorders, representing risk factors for mental health. The present study aims to analyze adverse childhood experiences, levels of resilience, early maladaptive cognitive schemas, and life history-based strategies among clinically healthy individuals and those diagnosed with psychopathological disorders. *Methods*: The study starts from the premise that adverse childhood experiences and maladaptive cognitive schemas will be more prevalent in the group of individuals with psychiatric disorders, while resilience will be lower in the psychopathological group. Furthermore, it is anticipated that the relationship between adverse childhood experiences and the onset of psychiatric problems is influenced by both internal mechanisms and protective factors. Specifically, it is expected that early maladaptive schemas will mediate this relationship, while resilience and life history-based strategies will attenuate the negative impact of adverse experiences, depending on the level at which these personal resources are present. The research included 106 Romanian participants, with an average age of 31.17 (SD = 13.06). *Results*: The results showed that adverse experiences and maladaptive cognitive schemas were significantly higher in the clinical group, F(104) = −6.97, *p* < 0.001. Additionally, resilience was significantly higher in the group of healthy individuals, F(104) = 8.76, *p* < 0.001. Psychiatric disorders were statistically significantly predicted by adverse childhood experiences (β = 2.46, *p* < 0.01). Regarding the mediation analysis of the relationship between adverse childhood experiences and psychiatric disorders, partial mediations were observed for the following variables: Emotional Deprivation, Social Isolation, Abandonment, and Impaired Self-Control. However, neither resilience nor life history-based strategies moderated the relationship between adverse childhood experiences and psychiatric disorders. *Conclusions*: The study’s results represent an important starting point for future research. Moreover, the current paper contributes to a better understanding of the presented concepts, offering significant implications for both theory and practice.

## 1. Introduction

Living in an unstable environment during childhood and adolescence can cause the formation and internalization of maladaptive cognitive patterns, which over time favor the somatization of unfavorable psychological factors into mental disorders [1,2]. Of course, a number of external factors also influence these outcomes. Internal factors such as resilience, coping styles, which are developed in response to positive or negative experiences, play a significant role [3,4]. Additionally, the environment of origin contributes to shaping these consequences. Thus, based on the above arguments, it is imperative to know these causes and consequences that can impact the individual in terms of mental health, with the aim of understanding, raising awareness, and preventing the elements that favor the triggering of such reactions in the body.

Children who grow up in adverse living conditions and environments encounter numerous challenges, which cause them to adapt to the dangers and barriers that stand in their way, involuntarily developing what we call the concept of resilience [4,5]. Education, parenting styles, and contextual factors are agents of resilience development [6,7]. Adverse living environments cause high levels of stress on the body, and the behavioral mobilizations and adaptations made to cope with these major stressors have negative effects on the well-being and functioning of the individual [8,9].

Evidence from meta-analyses linking adverse childhood experiences to an increased risk of mental disorders throughout life [10] shows that approximately 30% of all mental disorders can be attributed to these events [11]. Of these, 30% are cases of anxiety, 40% are cases of depression [12], and 67% are suicide attempts [13]. Childhood sexual abuse is also associated with a 13% risk of developing depression [14]. In a representative study conducted in the US, it was found that three out of five adults reported at least one adverse event in childhood, and a quarter of them experienced at least three such traumas [15].

Thus, given the high prevalence of adverse events in society [16] and their association with multiple psychiatric disorders, prevention and intervention measures should be considered to reduce the significant consequences of mental disorders and suicide.

Findings from multiple studies show that exposure to multiple adverse childhood experiences can have direct effects on mental health and well-being [17]. These adversities can include emotional abuse and neglect [18] negative life events, low levels of emotional resilience, traumatic experiences, and a lack of family problem-solving skills and hopefulness [9]. All of these factors can have a robust impact on drug use, alcohol use, depressed affect, and suicide attempts, which over time can contribute to the development of psychiatric disorders [17,19].

In Romania, according to data from studies conducted on representative samples, the most prevalent mental disorders among adults are anxiety disorders—of which specific phobias have a prevalence of 3.8%—and major depression, with a lifetime prevalence of 2.9%. At the same time, higher rates of anxiety disorders and depression were reported during the COVID-19 pandemic: 11.9% prevalence for panic disorder, 28.2% for generalized anxiety disorder, 26.8% for major depression, and 38% for post-traumatic stress disorder (PTSD) [20].

This study aims to investigate exposure to adverse events in childhood and the subsequent onset of psychiatric disorders. Through this approach, we want to demonstrate that the individual not only functions and survives in a certain environment, but is also constructed and shaped by it. In turn, the individual reconstructs it based on the contextual effects that have been directed at them, whether stable or unstable, through which they form a behavioral pattern, filtered through their own mind, as a reaction and direct effect of the variables that have shaped their personality.

In the following sections, we describe theoretical information, research variables, our methodology, present our findings, and then discuss their implications before drawing conclusions.

## 2. Conceptualization and Theoretical Aspects

### 2.1. Research Variables, Theoretical Argumentation, and Practical Implications

#### 2.1.1. Adverse Childhood Events and Psychiatric Disorders

Child maltreatment and related events are risk factors for mental health. Adverse childhood experiences are associated with forms of abuse (emotional, physical, sexual), neglect (emotional and physical), and living in households marked by stressors such as domestic violence, relationship difficulties (such as parental separation or divorce), excessive alcohol or drug use by family members, or criminal behavior [21,22].

Adverse childhood experiences are potentially traumatic and stressful experiences that occur during childhood or adolescence, affecting mental health and well-being [23,24]. They can manifest themselves through parental dysfunction, violence, socioeconomic adversity, and maltreatment.

Children or adolescents exposed to these events are at increased risk of developing behavioral problems or internalizing symptoms [25]. Adverse childhood events can also influence the severity of psychiatric disorders such as schizophrenia or bipolar disorder, as highlighted by Fisher & Hosang [21]. Numerous studies confirm that the presence of adverse events in childhood has a significant impact on Major Depressive Disorder, Anxiety disorders, dissociative disorders and substance abuse [26,27,28,29].

A large number of studies show that 50% of bipolar disorders are associated with a severe history of trauma or abuse during childhood [30,31,32,33].

Epidemiological and neurobiological studies highlight that adverse childhood events, such as physical or sexual abuse, are associated with brain dysfunction, which affects both physical and mental health [28,34] and also the long-term effect of childhood trauma determins inflammation and white matter disfunction in psychiatric disorders [33]. Thus, individuals exposed to adverse events are at increased risk of developing somatic and mental disorders throughout their lives. Functional changes have been identified in brain regions associated with stress and emotions, such as the anterior cingulate cortex, amygdala, and hippocampus [31,32]. Volumetric alterations in these brain areas have also been reported.

#### 2.1.2. Neuronal Changes

There is clear evidence that adverse childhood events and associated disorders are linked to long-lasting effects on the structure and functioning of neural circuits involved in stress regulation, such as the hippocampus, amygdala, or anterior cingulate cortex. Changes in stress sensitivity and emotion regulation have also been identified [35,36].

Many studies highlight reduced hippocampal volume in individuals who experienced adverse events in childhood compared to those who were not maltreated [28,31]. A greater reduction in hippocampal volume has also been observed in men than in women, suggesting that increased resilience in women may be associated with a protective effect of estrogen [37].

Amygdala volume was found to be increased in children raised in orphanages [38], children with mothers diagnosed with chronic depression [39], and adults who had dysfunctional attachment relationships in childhood [40]. In contrast, a reduction in amygdala volume has been found in adults later diagnosed with borderline personality disorder [41] and those with substance use disorders [42]. Amygdala hypertrophy may be associated with early exposure to emotional neglect, while decreased amygdala volume is more commonly reported in older adults with higher levels of psychopathology and a history of very severe abuse [40].

Women who were exposed to emotional abuse showed a reduction in the size of brain regions involved in self-awareness and self-evaluation, as highlighted by Herzog & Schmahl [19].

A series of studies have shown that exposure to certain specific adverse events selectively affects the sensory systems involved in the perception of experienced trauma [36]. For example, exposure to parental verbal abuse influences the arcuate fasciculus, a region that interconnects Broca’s area and Wernicke’s area [43], and exposure to domestic violence affects the inferior longitudinal fasciculus, which connects the visual system to the limbic system [44].

Thus, the amygdala performs an essential function, being involved in the detection and processing of stimuli associated with danger [45]. Amygdala hyperactivity has been linked to post-traumatic stress disorder (PTSD) [46], anxiety [47], and borderline personality disorder [48]. The insula has also been found to show increased activity in individuals exposed to strong emotional stimuli [49].

Adverse childhood events also affect psychosocial mechanisms that influence cognitive and affective processing, leading to increased attention to aversive stimuli [31], disrupting social interactions [50], and promoting aggressive behaviors [51]. The risk of developing psychiatric problems is higher in the case of depression, borderline personality disorder, post-traumatic stress disorder (PTSD), and substance use, as mentioned by Herzog & Schmahl [19].

The Diathesis-Stress Model expresses the idea that psychological disorders arise as a result of the interaction between vulnerability factors, such as biological and genetic predispositions, and stressful life experiences [52]. Psychiatric disorders can be triggered by the interaction between a diathesis (a genetic vulnerability or predisposition) and stressful life events [53,54]. The model specifies that the manifestation of a disorder depends on both the nature of the diathesis and the severity of the stressor to which the person is exposed.

Stressors that can contribute to the development of mental disorders include early trauma, severe childhood illness, sexual or physical abuse, prolonged unemployment, loss of a loved one, serious medical conditions, generational trauma, immigration and acculturation stressors, natural disasters, experiences of discrimination and community violence [33,55,56]. From this perspective, early adverse experiences can have a profound impact on the development of the brain and emotional regulation systems. They can increase long-term sensitivity to stress and affect the development of the central nervous system, including the neural connections involved in anxiety, impulse control, and behavioral regulation.

These experiences do not directly cause a disorder, but in the presence of a genetic predisposition (diathesis), they can trigger mental health problems such as depression, anxiety, personality disorders, or even psychosis. Studies support this relationship, demonstrating that the risk of developing mental disorders increases proportionally with the number and severity of adverse childhood experiences [55,57,58,59].

Thus, the diathesis-stress model provides a solid explanation for the causal relationship between adverse events and mental health, highlighting the importance of early intervention and psychosocial support for people at risk.

In this case, a very important aspect that can act as a buffer in the process of counteracting psychiatric symptoms is the individual’s accumulated internal resilience. Resilience can be conceptualized as an individual’s dynamic capacity to withstand or “recover from significant challenges that threaten their stability, viability, or development” [60]. More specifically, it is a positive adaptation or ability to maintain and regain well-being despite experiencing adversity and environmental challenges [61]. Resilience is a concept that develops on a continuum of life, which is acquired and refined by individuals who, over time, starting in childhood, successfully overcome difficult situations and strengthen their ability to further influence their environment [62]. The development of resilience involves personality traits that emerge in early childhood, coping strategies, and environmental factors [63]. Findings highlight it’s importance for prevention and health promotion, along with the moderating influence of resilience on the relation between ACE and mental health [64].

From a practical point of view, investigating this topic brings to the fore the aspect of confronting negative factors, which is an inevitable element. Those with a high level of internal resilience are much better prepared to cope with stressful events, having an improved quality of life, as cited by Good [65]. Thus, assimilating this information can contribute significantly to society in reshaping the perception of the concept of resilience, associated as an effect in one’s own development, following life’s adversities, through which people choose to confront the problem rather than avoid it [65]. In order to identify resilience in adult life, two conditions must be present: individuals must have experienced or been exposed to significantly risky events that increase the likelihood of negative development, and they must develop positive adaptation despite the risk to which they are exposed [6,60].

#### 2.1.3. Life History Strategy

According to Life History Strategy Theory [66], there is an evolutionary explanation for the link between adverse childhood events and the onset of psychiatric disorders. This theory argues that humans develop “fast” or “slow” life strategies depending on the environment in which they grow up. In a safe, stable, and predictable environment, children tend to develop a “slow” strategy, characterized by self-regulation, long-term investments in relationships, and better mental health. In contrast, in an adverse environment marked by insecurity, abuse, or neglect, the child learns—adaptively, but at a cost—to live “fast forward”: with impulsive reactions, seeking immediate rewards, and difficulties in regulating emotions. All of this can lead, over time, to increased vulnerability to psychiatric disorders such as anxiety, depression, obsessive-compulsive disorder, schizophrenia, personality disorders, or destructive behaviors. Therefore, from the perspective of life history strategies, the behaviors and psychological patterns observed in people who have had a childhood marked by adversity are not simply “dysfunctions,” but adaptations to an environment perceived as unstable and dangerous, which—in the absence of a change in context—can become disadvantageous in adult life.

The life history strategy is an evolutionary theory that describes the allocation of resources and energy within important life contexts, based on various compromises [67]. Taking into account environmental conditions, the strategies adopted in an individual’s evolution aim to maximize their fit and adaptation to society and life [68]. For example, individuals who develop in an unstable and unpredictable environment will develop traits consistent with the fast life strategy, associated with high reproduction rates, low parental investment, and short intervals between generations caused by immediate rapid reproduction at an early age [66]. In contrast, people who grow up in a stable, predictable environment tend to develop traits specific to the slow life strategy, which are associated with high parenting investment, low reproduction rates, and long intervals between generations [66]. Thus, the adoption of one of these strategies depends strictly on the characteristics of the environment during childhood. Environments characterized by harshness and unpredictability lead to the development of a fast life strategy [69], which is associated with early physical maturation and early childbearing [66]. This means that living in such an environment can cause the future adult to adopt coping strategies and resilience mechanisms that are not particularly beneficial. In contrast to fast life strategies, environments characterized by stability cause individuals to adopt a slow life strategy, where life expectancy is much longer, allowing them to invest in personal and social development and in their offspring [66,70].The characteristics of a slow life strategy are manifested through: long-term considerations in thinking and decision-making, high parental investment, monogamy, substantial social support, and adherence to social rules.

##### Strategies Based on Life History and Psychiatric Disorders–Relationships

From an evolutionary perspective, any behavior or trait that gives an organism an advantage in increasing its chances of survival and reproduction is considered adaptive. However, from a mental health perspective, some traits that are biologically adaptive may cause psychological distress or interpersonal difficulties [71]. What may be adaptive may not be considered desirable from a mental health perspective and could be viewed as symptoms of psychopathology [72,73].

Thus, although behaviors that promote a fast life strategy may be biologically adaptive, in the long term, from a social and mental well-being perspective, they are not necessarily desirable [73]. Psychopathological symptoms of fast life strategies can manifest themselves in the form of aggression, sexual promiscuity, reduced empathy, self-harm, risk-taking, disinhibition, and impulsivity [71,73].

Although aggression can be considered adaptive from the perspective of fast life strategies, from a mental health perspective, it is considered socially inappropriate, being a predictor of poor emotional and social development, isolation, and affective and personality disorders [74,75], aspects also cited by Hurst & Kavanagh [76]. The study by Hurst & Kavanagh showed that a fast life strategy is significantly associated with more psychopathological symptoms and more aggression. Thus, the study of these concepts provides insight into how life history-based strategies, such as adopting a fast life strategy, can develop psychiatric symptomatology, influencing psychological functioning and mental well-being. These aspects highlight and reinforce previous research in which the presence of an unstable environment affects social, emotional, and physical development [77,78].

Del Giudice reviewed the existing empirical evidence and categorized disorders associated with the fast or slow life spectrum. The disorders associated with fast life strategies were as follows: Autogenic Obsessive-Compulsive Disorder, Borderline Personality Disorder, Eating Disorders, Impulse Control and Conduct Disorders, Schizophrenia Spectrum, Alcohol Use Disorders. Disorders associated with slow life strategies were as follows: Depression, Eating Disorders (perfectionist and overcontrolled profiles), Reactive Obsessive-Compulsive Disorder, Alcohol Use Disorders [73].

Schizophrenia is associated with sexual promiscuity [79] and reduced long-term investment in romantic relationships [80]. Schizotypal traits are associated with high levels of aggression and low frustration tolerance. Research associates harsh living environments with an increased susceptibility to the risk of developing schizophrenia [81].

Borderline personality disorder is characterized by difficulties in emotional regulation, fear of abandonment, increased impulsivity, risk-taking, and unstable interpersonal relationships. There are studies that highlight the association of borderline disorder with a fast-paced lifestyle, through reduced parental investment, involvement in risky sexual activities, and adverse events experienced in childhood [82,83,84].

Depression is characterized by reduced motivation, decreased appetite or weight gain or loss, apathetic mood, fatigue or lack of energy, feelings of worthlessness or excessive guilt, inner emptiness, recurrent thoughts of death or suicidal ideation, agitation or psychomotor slowness, insomnia or hypersomnia. Depression may embody a fast-paced life strategy marked by disinhibition, high aggression, impulsivity, and low socioeconomic status [84,85,86].

Brune also stipulated the idea that psychiatric disorders can highlight a mix of fast and slow life strategies, being more of an interaction between them than a unipolar spectrum. Each disorder has a coordinate that presents both fast and slow traits, relative to strategies based on life history [82].

#### 2.1.4. Cognitive Schemas

Automatic thoughts are activated in response to certain triggering events, having the most accessible level of knowledge. Cognitive schemas operate at a broad level of generalization, supporting both automatic thoughts and intermediate beliefs; which are persistent over time and in various situations and contexts [87]. Beck described cognitive schemas as structures that encode and evaluate stimuli, helping individuals to give personal meaning to the surrounding reality, to interpret and organize information in order to guide and influence thinking and behavior.

Young introduces the term early maladaptive schemas and considers them to be cognitions about oneself and one’s relationships with others that develop in childhood, are elaborated throughout life, and remain stable throughout life, ensuring cognitive consistency [2]. He highlighted their role in chronic psychological stress, identifying a set of maladaptive schemas that can lead to self-destructive patterns of thinking and behavior [88]. The schemas begin to form as a result of the child’s relationships with their parents, who tell them who they are and what the world is like; through the lens of child-parent interaction, the child assimilates whether they are worthy of love, whether others are trustworthy, whether they are capable or safe, subsequently internalizing this set of cognitions and representations about themselves and the world, integrating them as fundamental truths according to which they will choose to act and think further in life [89].

##### Cognitive Schemas and Psychiatric Disorders–Relationships

Adverse events in childhood are considered risk factors for the development of symptoms specific to the schizophrenia spectrum; childhood trauma can cause psychotic symptoms [90]. Also, absence of child emotional resilience and lack of family problem-solving skills were significantly associated with depression [9].

Repeated exposure to traumatic events can impact a child’s emotional, behavioral, cognitive, social, and physiological functioning due to the brain’s malleability and sensitivity to early childhood experiences, which lead to hypersensitivity to stressors [91]. These aspects are also cited in the study by Bortolon, which highlights the idea that this hypersensitivity may increase the predisposition to psychiatric symptoms [90]. Birchwood [92] and Garety [93] suggested that adverse childhood events can lead to the development of negative schemas about oneself and the world, schemas involving vulnerability, subordination, and humiliation, which, in addition, facilitate the onset of psychotic symptoms in those with a genetic predisposition [94]. At the same time, cognitive schemas may even complicate the course of bipolar disorder development [95]. In his study Hawke respondents with bipolar disorder and anxiety reported cognitive schemas of Emotional Deprivation, Social Isolation, and Emotional Inhibition [94].

There are studies in the previous literature that confirm that adverse childhood events are related to a higher predisposition to psychiatric disorders than in people without a history of trauma, more specifically, 30% of all mental disorders are attributed to adverse childhood events [11]. In the study by Raj, a negative correlation was identified between adverse childhood events and psychological well-being, meaning that as adverse events increase in intensity, mental well-being decreases. This suggests that adverse childhood events can have long-lasting harmful effects on psychological health [96]. Their study also identified a negative association between automatic thoughts and psychological well-being. For example, a person with a higher level of automatic negative thoughts will have lower levels of psychological well-being.

The study by Gomez-Maquet highlights significantly higher rates of adverse childhood events in patients with a medical diagnosis of Major Depressive Disorder compared to the control group. Exposure to adversity in childhood is associated with an increased likelihood of developing depression; they conclude and confirm that adverse childhood events have a significant influence on Major Depressive Disorder [97]. At the same time, in terms of early cognitive schemas measured using the instrument developed by Young, patients with Major Depressive Disorder, compared to the control group, had significantly higher averages for each domain, with all early maladaptive schemas being positively correlated with depression [98].

In another study, a positive relationship was identified between adverse childhood events and early maladaptive schemas, where childhood abuse is closely linked to high rates of diagnosis with mental health disorders [28].

There is evidence that the earlier life strategies are adopted, the greater the association with psychopathological symptoms and aggression [76]. Lifestyle strategies can also influence mental health in many ways. Del Giudice reviewed the existing empirical evidence and categorized disorders associated with the fast or slow life spectrum [73]. Previous research highlights that environmental instability, characterized by a fast life strategy dominated by adverse events, negatively influences social and emotional development [99,100]. The results of the study by Hurst show that a fast life strategy significantly increases the chances of psychological dysfunction [76]. Similar findings were reported by Poole, whose results concluded that patients exposed to adverse events are at high risk for depression; participants who reported greater cumulative exposure to adverse events were more likely to report increased symptoms of depression. Also in their study, resilience moderated the relationship between adverse childhood events and depression [101].

Cumulative exposure to adverse childhood events associated with depression was significantly higher in individuals with low resilience. This means that high resilience values mitigate the effect between adverse childhood events and psychiatric disorders compared to low resilience values. Thus, a person with cumulative experiences of toxic events who also lacks adaptive mechanisms is much more susceptible to developing psychopathological problems.

### 2.2. Limitations of Previous Studies

As mentioned, in the literature, there are studies that support the influence of adverse events on the development of psychiatric disorders [96], or early maladaptive schemas positively correlated with psychopathology [97], cumulative exposure to adverse events in childhood associated with significantly higher psychiatric symptomatology in individuals with low resilience [101], and rapid life strategies significantly increase the chances of psychological dysfunction; the present study addressed all these variables in a single analysis and scientific investigation. In addition, our study considers a wide range of psychiatric disorders, unlike previous studies where research focused on the study of a specific disorder [97,101], where we will analyze whether cognitive schemas are predominant in most patients regardless of the specific disorder. We also analyzed the psychological determinants in patients admitted to acute psychiatric wards. The complex integration of several relevant psychological variables such as resilience, early maladaptive schemas, and life strategies. The paper presents the simultaneous testing of mediation and moderation roles, as few studies simultaneously address mediation and moderation in the same research, which brings a methodological advantage and allows us to know how early trauma influences mental health through cognitive schemas and under what conditions this influence is stronger or attenuated by analyzing resilience.

At the same time, the study is conducted on a Romanian sample, providing necessary information on the prevalence and impact of childhood events in Romania, with the adaptation of psychological constructs to a specific cultural context.

It is known from the study by Poole [101] that low levels of resilience were reported in patients diagnosed with psychiatric disorders, while in the study by Nunes [102], the comparison of resilience levels between different disorders showed that patients with major depression had lower levels of resilience compared to those diagnosed with bipolar disorder or schizophrenia. Also in their study, sociodemographic information was associated with resilience, where personal factors such as age, intelligence level, or education interfere with the level of resilience, which differs from one disorder to another.

Although previous studies have shown that there is a strong association between adverse childhood events and psychiatric disorders [1,4,6,8,9,64,103], the present study aims to use a comparison strategy between participants with a clinical diagnosis and those in the control group, represented by those without a diagnosis in terms of resilience, early maladaptive schemas, and life strategies addressed. Our research brings a new perspective, starting from the premise that living in an unstable environment, marked by adverse events such as abuse, neglect, parental separation, and family instability, can profoundly affect a child’s development, damaging their self-image, creating a distorted perception of the world and interpersonal relationships, and leading to beliefs of isolation, pessimism, shame, emotional deprivation, abandonment, low self-control, and poor emotional regulation. People with a history of adverse events and maladaptive patterns may develop a reduced ability to cope with stress, fewer effective strategies for adapting to stress, low tolerance, and a tendency to develop maladaptive mechanisms that favor the onset of mental health problems. In the long term, the interaction of these factors can create fertile ground for mental imbalance, leading to the onset of psychiatric disorders.

## 3. Materials and Methods

### 3.1. Research Design

The study aims to verify and test the differences between groups of people with psychiatric disorders and healthy individuals in terms of adverse childhood events, early maladaptive schemas, resilience, and life history-based strategies. The research also aims to investigate the potential mediating role of early maladaptive schemas in the relationship between adverse childhood events and psychiatric disorders, as well as the moderating role of resilience and life history-based strategies on the relationship between adverse childhood events and mental health disorders. Based on these premises, we will analyze the following hypotheses: adverse childhood events in the group of people with psychiatric disorders are significantly more numerous compared to the group of healthy people; early maladaptive schemas in the group of people with psychiatric disorders are significantly more numerous compared to the group of healthy people; resilience in the group of people with psychiatric disorders is significantly lower than resilience in the group of healthy people; adverse childhood events are a predictor of psychiatric disorders; early maladaptive schemas mediate the relationship between adverse childhood events and psychiatric problems; Resilience moderates the relationship between adverse childhood events and psychiatric disorders; more specifically, high resilience values will attenuate the relationship between adverse childhood events and psychiatric disorders compared to low resilience values. Life history-based strategies moderate the relationship between adverse childhood events and psychiatric problems.

The present study has a non-experimental cross-sectional design, with adverse childhood events as the independent variable, psychiatric disorders as the dependent variable, resilience and life history-based strategies as moderating variables, and early maladaptive schemas as mediating variables.

### 3.2. Ethics

From an ethical standpoint, all participants signed written informed consent forms before participating in the study. Participants were informed that participation was strictly voluntary and that they could withdraw from the process at any time during the research. All procedures were conducted in accordance with international ethical standards, including the Declaration of Helsinki and approved by the Institutional Review Board of Alexandru Obregia Clinical Hospital for Psychiatry. Participants data were anonymized, and confidentiality was strictly maintained throughout the study.

### 3.3. Participants and Data Collection

A total of 106 Romanian participants took part in this study, of whom 71 were women (67%) and 35 were men (33%). Of the total, 92 participants (86.8%) came from urban areas and 14 (13.2%) from rural areas. The overall mean age was 31.17 years (SD = 13.06). The sample was selected using convenience sampling, with participants recruited via email invitations and social media posts. Participants were divided into two groups. Inclusion in the two groups was based on a specific questionnaire with questions related to possible current or remitted psychiatric disorders. The control group consisted of students from the Faculty of Psychology and Education Sciences, and subjects were also recruited online by administering a questionnaire created in Google Forms and distributed on social media, Facebook groups, and Instagram. The experimental group consisted of subject with psychiatric disorders, recruited from inpatients admitted to the ‘Al. Obregia’ Hospital. In the control group, the exclusion criteria were the presence of any type of psychiatric disorder diagnosis and recurrent substance use. Another condition was a minimum age of 18 years. The group of patients with mental health problems consisted of 44 participants, aged between 21 and 76 years, with an average age of 36.75 years (SD = 16.16). The control group, consisting of 62 healthy participants, ranged in age from 18 to 51 years, with a mean age of 27.21 years (SD = 8.42).

### 3.4. Research Tools

Psychiatric disorders were assessed using a self-reported questionnaire that included specific questions such as: “Do you suffer from or have you received treatment for a psychiatric disorder?” and “Do you currently suffer from a specific diagnosed psychiatric disorder or have you suffered from one in the past, with the condition now in remission (e.g., depression, bipolar disorder, schizophrenia, PTSD, personality disorder, anxiety disorders, obsessive-compulsive disorder, dissociative disorders, somatic symptom disorders, eating disorders, substance use disorders)?” The answers were dichotomous: Yes or No.

Adverse childhood events were assessed using the Childhood Trauma Questionnaire—Short Form (CTQ-SF), developed by Bernstein and Fink [104,105]. It consists of 28 items and is designed to identify traumatic childhood experiences such as emotional, physical, and sexual abuse, as well as emotional and physical neglect. Of the 28 items, 25 are used to measure the five dimensions of maltreatment, while 3 additional items are included to detect tendencies to minimize or deny childhood trauma (e.g., “I had the best family in the world”). Responses are given on a 5-point Likert scale, ranging from 1—Never true to 5—Very often true. In the present study, the internal consistency coefficient of the scale (Cronbach’s α) for measuring adverse childhood events was α = 0.80, indicating good reliability of the instrument.

Life history-based strategies were assessed using the Mini-K Life History Strategy Short Form [106]. This consists of 20 items, a battery of cognitive and behavioral indicators of life strategy, adapted from various sources. The items measure individual differences, representing a single latent construct, the K factor; the items may indicate a “slow” or “fast” life strategy (“I would prefer to have multiple sexual relationships at the same time” or “I need to be very attached to someone before having sex”). A Likert scale was used to evaluate the items, with response options ranging from 1 (Strongly disagree), 2 (Disagree), 3 (Partially disagree), 4 (Don’t know/Not applicable), 5 (Partially agree), 6 (Agree), 7 (Strongly agree). The internal consistency coefficient resulting from the group of participants for measuring life strategies was α = 0.80. The total score is calculated, depending on the response to the Likert scale, as follows: −3 for total disagreement, −2 for disagreement, −1 for partial disagreement, 0 for don’t know/not applicable, +1 for partial agreement, +2 for agreement, +3 for total agreement.

Resilience was assessed using the Connor-Davidson Resilience Scale (CD-RISC) questionnaire [107]. It consists of 25 questions designed to assess adaptability to change, problem-solving skills, taking responsibility for coping with stress, the presence of stable emotional bonds, the ability to endure stress, strategies for achieving goals, and self-confidence (“When things seem hopeless, I don’t give up”). A Likert scale was used to evaluate the items, with response options ranging from 1 (Not at all true), 2 (Rarely), 3 (Sometimes true), 4 (Often true), 5 (Always, most of the time). Respondents will report on how they felt in the last month. High scores indicate good resilience and adaptability.

Early cognitive schemas were assessed using the Young Schema Questionnaire—Short Form 3 (YSQ-S3) [2], adapted to the Romanian population [108]. The research questionnaire included scales for Social Isolation, Emotional Deprivation, Abandonment, and Self-Control. Respondents will rate each item on a 6-point Likert scale, where: 1 = totally untrue for me, 2 = mostly untrue for me, 3 = more false than true, 4 = more true than false, 5 = mostly true for me, 6 = describes me perfectly. High scores indicate/provide relevant information about the beliefs and schemas of the person “If I make a mistake, I deserve to be punished.”

### 3.5. Analysis

Pearson correlation analysis in SPSS was used to evaluate descriptive statistics and correlations between research variables. The moderating effects of strategies based on life history, resilience, and the mediating effect of early maladaptive schemas were statistically analyzed using moderation and mediation analysis. Statistical analyses were performed using SPSS version 28.0.0 and JAMOVI version 2.6.44 software.

To ensure that we had a reasonable probability of correctly rejecting the null hypothesis and to determine the extent to which the acceptance of a null hypothesis was not due to an effect size that was too small, in order to avoid type II error, we calculated the power analysis using the G*Power platform version 3.1.9.7.

Descriptive analysis was used for the variables of interest to provide an overview of the data. Correlation analysis between variables using Pearson’s correlation was used to verify linear or monotonic relationships between adverse childhood events, psychiatric disorders, early maladaptive schemas, resilience, and life history-based strategies.

To compare scores on variables of interest between the control and experimental groups, the t-test for independent samples was used. The t-test for independent samples was used to compare scores on variables of interest (e.g., psychiatric disorders) between groups (e.g., individuals with and without adverse childhood events).

Binary logistic regression analysis was used to identify the predictability of psychiatric disorders based on adverse childhood events.

## 4. Results

The statistical analysis for this study was structured to examine the relationship between adverse childhood experiences (ACEs), resilience, cognitive-emotional schemas, and the presence of psychiatric disorders. The plan was designed to evaluate group differences, test associations among key variables, and build predictive and mediational models to clarify the underlying mechanisms.

The first stage consisted of descriptive statistics to summarize the characteristics of the sample and the main research variables. Means, standard deviations, and distributional indices were calculated. Pearson correlation coefficients were then employed to assess the associations between ACEs, resilience, life history strategies (LHT), and maladaptive cognitive schemas (social isolation, emotional deprivation, abandonment, and impaired self-control). Pearson’s correlation was selected because it is appropriate for continuous variables and allows for the detection of both positive and negative linear relationships. These correlations served as a foundation for subsequent regression and mediation analyses by identifying which constructs were interrelated and potentially important as predictors or mediators.

To test the central hypotheses regarding group differences between participants with psychiatric diagnoses and controls, independent-sample t-tests were conducted. This test was chosen because it is well-suited to compare means between two independent groups on continuous outcome measures, assuming normality and homogeneity of variances. Each t-test examined whether the groups differed significantly in levels of ACEs, resilience, and cognitive schemas. Effect sizes (Cohen’s d) were computed to complement statistical significance and assess the magnitude of group differences. These comparisons established the clinical relevance of the constructs before modeling predictive pathways.

A binary logistic regression analysis was performed to determine the predictive power of ACEs on psychiatric disorders (coded as presence vs. absence). Logistic regression was selected because the dependent variable was categorical (diagnosis vs. control). Variable inclusion was theory-driven: ACEs were introduced as the primary predictor based on the hypothesis that childhood adversity increases vulnerability to psychopathology. The model was evaluated using odds ratios (ORs), regression coefficients (β), and explained variance (Nagelkerke R^2^). The results showed that ACEs significantly predicted the likelihood of psychiatric disorders, with each additional ACE score increasing the odds of psychiatric diagnosis by 71%.

Potential confounders (e.g., age, gender, resilience, and maladaptive schemas) were not entered simultaneously in this initial regression but were instead examined through subsequent mediation and moderation models. This stepwise design minimized overfitting while isolating the direct effect of ACEs.

To test indirect pathways, mediation models were estimated for social isolation, emotional deprivation, abandonment, and self-control. Each variable was modeled as a potential mediator in the relationship between ACEs and psychiatric disorder status. Mediation was assessed by estimating direct, indirect, and total effects using path coefficients, standard errors, and z-tests. Bootstrapping techniques were applied to increase robustness of the indirect effect estimates. The rationale for mediation testing was grounded in cognitive schema theory, which posits that early adversity shapes maladaptive cognitive-emotional patterns, which in turn contribute to psychopathology. Results supported partial mediation across all four schemas, suggesting that these maladaptive patterns partially transmit the effect of ACEs on psychiatric outcomes.

Finally, resilience and life history strategies (LHT) were examined as moderators of the ACE–psychiatric disorder link. Interaction terms (ACE × Resilience and ACE × LHT) were entered into logistic regression models. Moderation was tested to explore whether protective or adaptive strategies could buffer the negative impact of childhood adversity. The rationale was based on resilience frameworks suggesting differential susceptibility to adversity. However, neither resilience nor LHT significantly moderated the relationship, indicating that the association between ACEs and psychiatric disorders is relatively robust across these individual differences.

Across analyses, assumptions were addressed as follows: normality and homogeneity were checked prior to t-tests; linearity in the logit was assumed for logistic regression, consistent with the theoretical justification for ACEs as a continuous risk factor; multicollinearity was minimized by introducing predictors separately or within mediation structures rather than in a single regression block; independence of observations was guaranteed by study design; Sample size adequacy was considered acceptable given the logistic regression rule of thumb (>10 events per predictor).

In this way we have had a systematic approach, beginning with descriptive and inferential analyses, progressing to regression modeling, and extending into mediation and moderation frameworks. It ensures both the detection of group-level effects and the exploration of underlying mechanisms, while adhering to statistical and theoretical rigor.

Table 1 shows the means and standard deviations for the research variables as well as the indicators referring to the distribution shape. The correlations between the research variables are also reported.

In our sample, statistically significant positive associations were observed among several variables. Higher Adverse Childhood Experiences (ACE) scores were associated with higher resilience (r = 0.62, *p* < 0.01), as well as with Life History Theory (LHT) measures (r = 0.62, *p* < 0.01). LHT and resilience were also strongly positively correlated (r = 0.73, *p* < 0.01). ACE scores were positively associated with social isolation (r = 0.51, *p* < 0.01), emotional deprivation (r = 0.60, *p* < 0.01), and feelings of abandonment (r = 0.38, *p* < 0.01). Emotional deprivation and social isolation were strongly correlated (r = 0.77, *p* < 0.01), and abandonment was positively associated with both social isolation and emotional deprivation (r = 0.67, *p* < 0.01).

Self-control was positively associated with ACE (r = 0.33, *p* < 0.01), social isolation (r = 0.64, *p* < 0.01), emotional deprivation (r = 0.57, *p* < 0.01), and abandonment (r = 0.72, *p* < 0.01). These results suggest that higher levels of adverse experiences in childhood are related to greater social and emotional difficulties in our sample, while also being linked to higher self-reported resilience and self-control. This suggests a complex relationship in which early adversity is associated with both greater vulnerability (more social and emotional difficulties) and certain adaptive strengths (higher resilience and self-control).

At the same time, several statistically significant negative relationships were observed in our sample. Social isolation was negatively associated with resilience (r = −0.44, *p* < 0.01) and Life History Theory (LHT) measures (r = −0.46, *p* < 0.01). Emotional deprivation also showed significant negative correlations with resilience (r = −0.43, *p* < 0.01) and LHT (r = −0.52, *p* < 0.01). Feelings of abandonment were negatively associated with resilience (r = −0.40, *p* < 0.01) and LHT (r = −0.29, *p* < 0.01). Additionally, self-control was negatively correlated with both resilience (r = −0.45, *p* < 0.01) and LHT (r = −0.29, *p* < 0.01). These findings indicate that higher levels of social and emotional difficulties in our sample are related to lower resilience and less adaptive patterns according to LHT measures (Table 2). In other words, as social and emotional difficulties increased, both resilience and adaptive behaviors decreased.

Adverse childhood events were significantly higher in the experimental group (M = 2.49, SD = 0.55) compared to the control group (M = 1.79, SD = 0.48), with the model being significant F(104) = −6.97, *p* < 0.001, 95% CI = [−2.0, −1.04]. Resilience was significantly higher in the control group (M = 3.79, SD = 0.60) compared to the group of individuals with a psychiatric diagnosis (M = 2.77, SD = 0.57), the model being significant F(104) = 8.76, *p* < 0.001, 95% CI = [0.79, 1.25]. Social isolation was significantly higher in the group of people with psychiatric diagnoses (M = 3.90, SD = 1.24) compared to the control group (M = 2.36, SD = 1.25), where F(104) = −6.22, *p* < 0.001, 95% CI [−2.0, −1.04]. Emotional deprivation was significantly higher in the group of people with psychiatric diagnoses (M = 4.07, SD = 1.19) compared to the control group (M = 2.58, SD = 1.27), where F(104) = −6.08, *p* < 0.001, 95% CI [−1.97, −1.00]. Abandonment was significantly higher in the group of individuals with a psychiatric diagnosis (M = 3.77, SD = 1.16) compared to the control group (M = 2.52, SD = 1.33), where F(104) = −4.99, *p* < 0.001, 95% CI [−1.74, −0.75]. The cognitive schema involving self-control was significantly higher in the group of people with a psychiatric diagnosis (M = 3.62, SD = 1.01) compared to the control group (M = 2.70, SD = 0.95), where F(104) = −4.71, *p* < 0.001, 95% CI [−1.29, −0.52]. Adverse childhood experiences (ACEs) are more common in the group of people with psychiatric diagnoses, with a significant difference between the two groups and a very large effect size (d = 1.37). Resilience is more common in the control group, with a significant difference between the two groups and a very large effect size (d = 1.74). Overall, individuals with psychiatric diagnoses reported significantly higher levels of adverse childhood experiences, social isolation, emotional deprivation, abandonment, and self-control difficulties, while resilience was significantly higher in the control group.

Table 3 presents the results of the logistic regression analysis. Adverse childhood experiences (ACEs) were introduced as a predictor for the presence of psychiatric disorders. With regard to the prediction of psychiatric disorders, the model is statistically significant with β = 2.46, *p* < 0.01, predicting 40% of the variance. Therefore, psychiatric disorders are statistically significantly predicted by adverse childhood experiences (β = 2.46, *p* < 0.01). Furthermore, each additional point in adverse childhood experiences increases the likelihood of predisposition to psychiatric disorders by 71%. These results suggest that individuals with higher exposure to adverse experiences in childhood are at substantially greater risk for psychiatric disorders, highlighting the strong influence of early life stressors on mental health outcomes.

Taking into account adverse childhood events, their effect predicts social isolation (β = 1.22, *p* < 0.001). Social isolation predicts the effect on the group (β = 0.10, *p* < 0.001). ACE positively predicts psychiatric disorders (β = 0.45, *p* < 0.001). Controlling for social isolation, ACE showed a direct effect on the group (β = 0.32, *p* < 0.001), achieving partial mediation. The effect of ACE on psychiatric problems through social isolation is statistically significant (β = 0.13, *p* = 0.002). Table 4 and Table 5 displays these results.

Taking into account adverse childhood events, their effect predicts Emotional Deprivation (β = 1.41, *p* < 0.001). Emotional Deprivation predicts the effect on the group (β = 0.09, *p* = 0.005). ACE positively predicts psychiatric disorders (β = 0.45, *p* < 0.001). Controlling for Emotional Deprivation, ACE showed a direct effect on the group (β = 0.32, *p* = 0.008), suggesting partial mediation. Table 6 and Table 7 displays these results.

Taking into account adverse childhood events, their effect predicts abandonment (β = 0.87, *p* < 0.001). ACEs positively predict psychiatric disorders (β = 0.45, *p* < 0.001). Controlling for Abandonment, ACE showed a significant direct effect on the group (β = 0.37, *p* < 0.001), suggesting partial mediation. Table 8 and Table 9 displays these results.

Taking into account adverse childhood events, their effect predicts self-control (β = 0.58, *p* < 0.001). ACE positively predicts psychiatric disorders (β = 0.45, *p* < 0.001). Controlling for Self-Control, ACE showed a significant direct effect on the group (β = 0.38, *p* < 0.001), suggesting partial mediation. Table 10 and Table 11 displays these results.

Part of these effects occur because ACEs contribute to the development of maladaptive cognitive and behavioral patterns, such as emotional deprivation, social isolation, abandonment, and reduced self-control, which in turn elevate the risk for psychiatric problems. However, even in the absence of these maladaptive schemas, ACEs retain a direct impact on psychiatric risk, highlighting the enduring influence of early adversity on mental health outcomes.

Resilience does not moderate the relationship between ACEs and psychiatric disorders. The interaction variable between ACEs and resilience does not have a significant effect on psychiatric disorders (B = 1.04, *p* = 0.41). Life history-based strategies do not moderate the relationship between ACEs and psychiatric disorders. The interaction variable between ACEs and life history-based strategies does not have a statistically significant effect on psychiatric disorders (B = −0.16, *p* = 0.80). See Table 12.

Resilience and life history-based strategies do not moderate the effect of adverse childhood experiences (ACEs) on psychiatric disorders. This suggests that the severity and accumulation of ACEs can overwhelm protective factors, leaving individuals vulnerable to psychiatric problems. Similarly, life history-based strategies are largely shaped by environmental context and internalized by the individual rather than actively altering the effect of ACEs.

## 5. Discussion

### 5.1. Theoretical and Practical Implications

The study examined differences between individuals with psychiatric disorders and those without a psychopathological history in terms of exposure to adverse events in childhood. The aim is to investigate the effects of resilience variables, life history-based strategies, and maladaptive cognitive schemas. It is assumed that there will be more adverse events in the group of people with a diagnosis and more maladaptive cognitive schemas. We also consider that resilience in the group of people with psychiatric disorders will be significantly lower than in the control group. At the same time, the study analyses whether early maladaptive schemas can explain the link between childhood trauma and mental disorders, and whether resilience and life strategies can influence this relationship.

Adverse childhood events were introduced as a predictor for the presence of psychiatric disorders. Based on the results, psychiatric disorders are statistically significantly predicted by adverse events, predicting 40% of the variance, which confirms our hypothesis. This result is consistent with previous research showing an association between adverse childhood events and an increased risk of mental disorders throughout life [6,9,17,29,33,109,110], where approximately 30% of all mental disorders can be attributed to these events [11].

It has also been shown that adverse childhood events in the group of people with psychiatric disorders are significantly more numerous compared to the group of healthy people, confirming the hypothesis. Adverse childhood events are more common in the group of people with a psychiatric diagnosis, with a significant difference between the two groups and a very large effect size (d = 1.37). At the same time, resilience was also significantly lower in the group of people with psychiatric disorders compared to the control group, thus confirming the previously formulated hypothesis. Resilience is more common in the control group, with a significant difference between the two groups and a very large effect size (d = 1.74). This result is consistent with previous research [111], where higher levels of resilience are related to fewer mental health problems and those who were exposed to higher levels of adversity had fewer protective factors [4]. Likewise, ACE were associated with less adaptive emotion regulation [112,113,114,115] which in turn dysfunctional regulation was associated with mental and physical health problems [109]. In Brodbeck study was also suggested that emotion regulation skills also affect the resilience of a person [116].

At the same time, maladaptive cognitive schemas such as Social Isolation, Emotional Deprivation, Abandonment, and Self-Control were significantly higher in the group of people with psychiatric diagnoses, once again confirming the hypothesis. This attests to the process by which cognitive schemas frequently develop as a result of early traumatic experiences or the failure to meet basic, fundamental needs [117] These crystallize and internalize during childhood and become stable and rigid filters through which the individual interprets reality, an aspect also confirmed by Hawke and Young [2,94]. Repeated exposure to trauma thus impacts emotional, behavioral, social, and physiological functioning, and due to the malleability of the brain and sensitivity to early childhood experiences, it leads to hypersensitivity towards the subsequent internalization of a set of cognitions and representations about oneself and the world, which will later be integrated as fundamental truths according to which the person will choose to act and think further in life [90,92,118]. These results suggest that the more adverse events were experienced in childhood, the more pronounced the dysfunctional metacognitive beliefs will be.

Regarding the mediation analysis in the relationship between adverse childhood events and psychiatric disorders, partial mediations of the variables Emotional Deprivation, Social Isolation, Abandonment, and Self-Control were obtained, confirming our hypothesis. This means that adverse childhood events independently increase the risk for psychiatric disorders. Part of this effect is due to the fact that adverse childhood events lead to the maladaptive cognitive schemas investigated, which in turn increase the risk for the development of psychiatric disorders. But even without these maladaptive cognitive schemas, adverse childhood events have their own direct impact on the risk of psychiatric disorders.

Both resilience and life history-based strategies do not moderate the relationship between adverse childhood events and psychiatric disorders. The reasoning behind these results stems from the fact that the severity and accumulation of adverse events can outweigh the protective effects of resilience, with adverse childhood events having a profound and lasting impact on mental and neuropsychological development, influencing how individuals perceive the world [2,25,89,91]. At the same time, another argument refers to the fact that even if a person develops a certain level of resilience, it may not be strong enough to completely compensate for the traumatic effects of adverse events, especially in cumulative cases [33,119,120]. This is evidenced by the imprint of trauma on the structure and functioning of the neural circuits involved in stress regulation, leading to changes in stress sensitivity and emotion regulation [35,36].

Contrary to these findings, Good argues that individuals with high levels of internal resilience are much better equipped to cope with stressful events and enjoy an improved quality of life [65,121]. However, as exposure to repeated adverse events affects the sensory systems involved in the perception of experienced trauma [36], the individuals concerned will automatically have limited resources in the process of self-awareness and self-assessment [19].

At the same time, including the theory of strategies based on life history highlights the fact that living in an environment characterized by harshness and unpredictability can cause the future adult to adopt coping strategies and resilience mechanisms that are not exactly beneficial [17,69].

Thus, according to the above results, resilience between adverse events and psychopathology may be a mediating rather than a moderating variable, where adverse events contribute to psychiatric disorders through the development of maladaptive cognitive schemas. In this case, resilience may influence overall functioning, but it does not directly block the effect of adverse events on the risk of psychiatric disorders.

Therefore, our results indicate that childhood trauma has a robust and direct impact on the risk of psychopathology, and resilience fails to significantly mitigate this link, suggesting the need for early intervention in restructuring maladaptive cognitive schemas.

As for strategies based on life history, the result shows that we cannot choose the strategy, the individual internalizes it according to the environment, so it cannot be perceived as a variable that “interrupts” the effect of adverse childhood events, but rather one that mediates the effect.

Based on our results, the research presents important practical implications that highlight the need for screening for childhood trauma in clinical assessments, information that is imperative to include in order to apply specific interventions to particular cases in psychotherapy. Furthermore, investigating early maladaptive schemas aids the process of cognitive restructuring in therapy, which in turn highlights that intervention on schemas can reduce the long-term effect of childhood trauma; all these results have a dependent effect on each other, acting bidirectionally.

Prevention programs can be implemented among young people that integrate the enhancement of psychological resources. It helps in the development of early screening tools for children in at-risk environments.

From a theoretical point of view, the concept of psychological resources is integrated, supporting stress-vulnerability models, maladaptive cognitive schemas, and quick life strategies in explaining how individuals respond to adversity, confirming the explanatory value of cognitive schemas in the process of internalizing adverse events. At the same time, it confirms the predictive nature of early traumatic experiences in the onset of cognitive dysfunction.

In conclusion, the results obtained outline a complex model in which adverse childhood events affect psychological health, and the resulting implications can guide future research, raise awareness among the population, and call for standardized clinical practice by personalizing psychological interventions and focusing on prevention. The results provide a solid explanation for the causal relationship between adverse events and mental health, highlighting the importance of early intervention and psychosocial support for people at risk.

### 5.2. Limitations

A significant limitation of the study concerns the small number of participants, which ensures the consistency and validity of the results; the experimental group had 45 participants, offering more variability between people, which means that natural differences between individuals can significantly influence the results.

Another important limitation to note refers to the participants targeted, namely the inclusion of individuals under psychiatric care in a psychiatric ward, in a context where active symptoms can significantly influence the accuracy of self-reports. This can lead to cognitive distortions, perception disorders, negative affect, and memory disorders, where the variables investigated may be perceived and reported through a distorted filter specific to the clinical condition. Therefore, the generalization of the results is limited, as the study population does not include individuals with symptoms in remission or outside the axis of the exacerbated psychiatric system.

Also, all data from the control group were collected through an online questionnaire, through self-reporting, so the information provided and the answers could have been influenced by social desirability. Given the large number of questions in the questionnaire, the present study did not measure the participants’ predisposition to boredom.

## 6. Conclusions

Our findings consolidate and extend the actual scientific evidence base by demonstrating, within a Romanian mixed clinical–community sample, that adverse childhood experiences exert a large, transdiagnostic effect on psychiatric status that persists even after accounting for core cognitive-emotional mechanisms. Early maladaptive schemas—social isolation, emotional deprivation, abandonment, and impaired self-control—partially mediate the ACE–psychopathology link, identifying concrete, modifiable targets for schema-focused interventions in acute psychiatric care. Importantly, we tested mediation and moderation simultaneously and found that neither resilience nor life-history–based strategies attenuated the ACE–disorder association in this cohort. This dual null moderation is informative: it suggests that, at least in acutely ill settings, the cumulative neurocognitive and relational imprint of adversity may overshadow the buffering influence of generalized resilience or strategic life-history orientations as they are commonly measured. Practically, this argues for prioritizing early adversity screening and schema-level formulation in routine assessments, and for embedding cognitive-schema restructuring alongside resilience-building, rather than relying on resilience alone to offset risk. The study also contributes cultural context by providing estimates and mechanisms in a Romanian sample, a population under-represented in ACE research, supporting the external validity of ACE effects beyond frequently studied Anglophone cohorts. Methodologically, we used a unified framework spanning group differences, predictive modeling, and path analysis to map both the magnitude (very large effect sizes for ACE and resilience group differences) and the routes (partial schema mediation) by which adversity confers risk. Future longitudinal work should test whether disorder-specific schema profiles show differential responsiveness to targeted therapy and whether more granular, domain-specific measures of resilience (e.g., social-ecological vs. individual traits) reveal conditional buffering not captured here. Concluding, this study underscores that what is most actionable in ACE-informed psychiatry may be the identification and modification of entrenched schemas through which adversity becomes psychopathology—an approach that is feasible, mechanism-based, and directly aligned with our data.

## Figures and Tables

**Table 1:** Descriptive statistics and Pearson correlations between research variables.

Variable	1	2	3	4	5	6	7	*M* (SD)
1. ACE	-							2.07 (0.61)
2. Resilience	0.62 **	-						3.36 (0.77)
3. LHT	0.62**	0.73 **	-					4.37 (0.98)
4. Social Isolation	0.51 **	−0.44 **	−0.46 **	-				3.00 (1.45)
5. Emotional Deprivation	0.60 **	−0.43 **	−0.52 **	0.77 **	-			3.20 (1.44)
6. Abandonment	0.38 **	−0.40 **	−0.29 **	0.67 **	0.67 **	-		3.04 (1.40)
7. Self-control	0.33 **	−0.45 **	−0.29 **	0.64 **	0.57 **	0.72 **	-	3.08 (1.07)

** *p* < 0.01.

**Table 2:** Means, standard deviations, and t-test results for research hypotheses.

ACE	Subjects Without Psychiatric Diagnosis (Control Group)	Subjects with Psychiatric Diagnoses (Experimental Group)	*t*
	*M* (*SD*)	*M* (*SD*)	
ACE	1.79 (0.48)	2.49 (0.55)	−6.97 **
Resilience	3.79 (0.60)	2.77 (0.57)	8.76 **
Social Isolation	2.36 (1.25)	3.90 (1.24)	−6.22 **
Emotional Deprivation	2.58 (1.27)	4.07 (1.19)	−6.08 **
Abandonment	2.52 (1.33)	3.77 (1.16)	−4.99 **
Self-control	2.70 (0.95)	3.62 (1.01)	−4.71 **

** = *p* < 0.01.

**Table 3:** Logistic regression for the predictive power of adverse childhood events on psychiatric disorders.

Independent Variable	Psychiatric Disorders		
	β	R^2^	OR xp(B)
ACE	2.46 **	0.40 **	0.71

** = *p* < 0.01

**Table 4:** Mediation Analysis for Social Isolation.

Effect	Estimate	SE	Z	*p*
Indirect	0.130	0.0424	3.07	0.002
Direct	0.325	0.0714	4.55	<0.001
Total	0.455	0.0647	7.04	<0.001

**Table 5:** Path Estimate.

			Estimate	SE	Z	*p*
ACE	→	Social Isolation	1.224	0.1976	6.19	<0.001
Social Isolation	→	Group	0.106	0.0301	3.54	<0.001
ACE	→	Group	0.325	0.0714	4.55	<0.001

**Table 6:** Mediation analysis for Emotional Deprivation.

Effect	Estimate	SE	Z	*p*
Indirect	0.131	0.0499	2.63	0.008
Direct	0.324	0.0781	4.15	<0.001
Total	0.455	0.0647	7.04	<0.001

**Table 7:** Path Estimates.

			Estimate	SE	Z	*p*
ACE	→	Emotional Deprivation	1.410	0.1823	7.74	<0.001
Emotional Deprivation	→	Group	0.093	0.0333	2.80	0.005
ACE	→	Group	0.324	0.0782	4.15	<0.001

**Table 8:** Mediation analysis for Abandonment.

Effect	Estimate	SE	Z	*p*
Indirect	0.081	0.0319	2.54	0.011
Direct	0.374	0.0670	5.59	<0.001
Total	0.455	0.0647	7.04	<0.001

**Table 9:** Path Estimates.

			Estimate	SE	Z	*p*
ACE	→	Abandonment	0.8789	0.2055	4.28	<0.001
Abandonment	→	Group	0.0922	0.0292	3.15	0.002
ACE	→	Group	0.3743	0.0670	5.59	<0.001

**Table 10:** Mediation analysis for Self-control.

Effect	Estimate	SE	Z	*p*
Indirect	0.069	0.0290	2.40	0.016
Direct	0.385	0.0654	5.90	<0.001
Total	0.455	0.0647	7.04	<0.001

**Table 11:** Path Estimates.

			Estimate	SE	Z	*p*
ACE	→	Self-control	0.580	0.1610	3.60	<0.001
Self-control	→	Group	0.120	0.0372	3.23	0.001
ACE	→	Group	0.386	0.0654	5.90	<0.001

**Table 12:** Moderating effect of Resilience and Life History-Based Strategies on the relationship between ACE and Psychiatric Disorders.

Dependent Variable	Predictor	*B* (*SE*)	*z*
Psychiatric Disorders	ACE	−2.13 (3.97)	−0.53
	Resilience	−4.76 (2.90)	−1.64
	ACE xResilience	1.04 (1.28)	0.81
Psychiatric Disorders	ACE	−1.06 (2.75)	−0.38
	LHT	1.08 (1.37)	0.43
	ACE x LHT	−0.16 (0.64)	−0.25

## Data Availability

The original contributions presented in this study are included in the article. Further inquiries can be directed to the corresponding author(s).
